# Tetra­kis[1-phenyl-3-(1*H*-1,2,4-triazol-1-yl-κ*N* ^4^)propan-1-one]bis­(thio­cyanato-κ*N*)manganese(II)

**DOI:** 10.1107/S1600536810047112

**Published:** 2010-11-20

**Authors:** Hua Cai, Ying Guo, Jian-Gang Li

**Affiliations:** a College of Science, Civil Aviation University of China, Tianjin 300300, People’s Republic of China

## Abstract

In the mononuclear title complex, [Mn(NCS)_2_(C_11_H_11_N_3_O)_4_], the Mn^II^ atom, lying on an inversion center, is coordinated by two monodentate thio­cyanate anions and four monodentate 1-phenyl-3-(1*H*-1,2,4-triazol-1-yl)propan-1-one ligands in a distorted octa­hedral geometry. Each complex mol­ecule is linked to four neighboring ones by weak C—H⋯N and C—H⋯S hydrogen bonds, forming a two-dimensional sheet parallel to (001).

## Related literature

For general background to self-assembly of supra­molecular systems, see: Beatty (2003 ▶); Braga *et al.* (2003 ▶). For a related structure, see: Guo & Cai (2007 ▶).
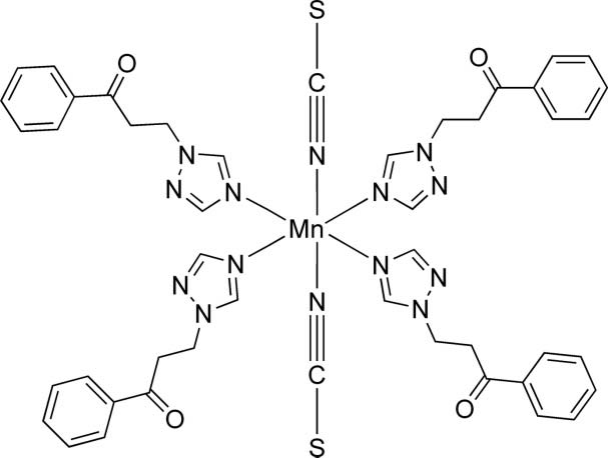


## Experimental

### 

#### Crystal data

[Mn(NCS)_2_(C_11_H_11_N_3_O)_4_]
*M* *_r_* = 976.03
Triclinic, 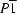

*a* = 7.9326 (17) Å
*b* = 11.845 (3) Å
*c* = 13.740 (3) Å
α = 69.240 (3)°
β = 75.417 (3)°
γ = 81.686 (3)°
*V* = 1166.1 (5) Å^3^
*Z* = 1
Mo *K*α radiation
μ = 0.43 mm^−1^
*T* = 293 K
0.20 × 0.18 × 0.14 mm

#### Data collection

Bruker APEXII CCD diffractometer
Absorption correction: multi-scan (*SADABS*; Sheldrick, 1996 ▶) *T*_min_ = 0.919, *T*_max_ = 0.942
6410 measured reflections
4075 independent reflections
2840 reflections with *I* > 2σ(*I*)
*R*_int_ = 0.021

#### Refinement

*R*[*F* ^2^ > 2σ(*F* ^2^)] = 0.038
*wR*(*F* ^2^) = 0.089
*S* = 1.06
4075 reflections
304 parameters
H-atom parameters constrained
Δρ_max_ = 0.19 e Å^−3^
Δρ_min_ = −0.23 e Å^−3^

### 

Data collection: *APEX2* (Bruker, 2007 ▶); cell refinement: *SAINT* (Bruker, 2007 ▶); data reduction: *SAINT*; program(s) used to solve structure: *SHELXS97* (Sheldrick, 2008 ▶); program(s) used to refine structure: *SHELXL97* (Sheldrick, 2008 ▶); molecular graphics: *SHELXTL* (Sheldrick, 2008 ▶) and *DIAMOND* (Brandenburg & Berndt, 1999 ▶); software used to prepare material for publication: *SHELXTL*.

## Supplementary Material

Crystal structure: contains datablocks global, I. DOI: 10.1107/S1600536810047112/hy2378sup1.cif

Structure factors: contains datablocks I. DOI: 10.1107/S1600536810047112/hy2378Isup2.hkl

Additional supplementary materials:  crystallographic information; 3D view; checkCIF report

## Figures and Tables

**Table 1:** Hydrogen-bond geometry (Å, °)

*D*—H⋯*A*	*D*—H	H⋯*A*	*D*⋯*A*	*D*—H⋯*A*
C12—H12⋯N2^i^	0.93	2.62	3.436 (3)	146
C18—H18⋯S1^ii^	0.93	2.82	3.725 (3)	164

Symmetry codes: (i) 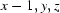; (ii) 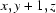.

**Fig. 1.**
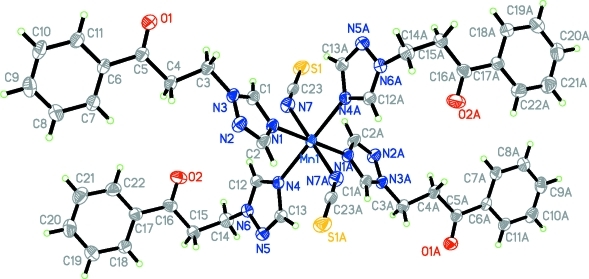
The molecular structure of the title compound, showing the 30% probability ellipsoids. [Symmetry code: (A) -x, -y, 2-z.]

**Fig. 2.**
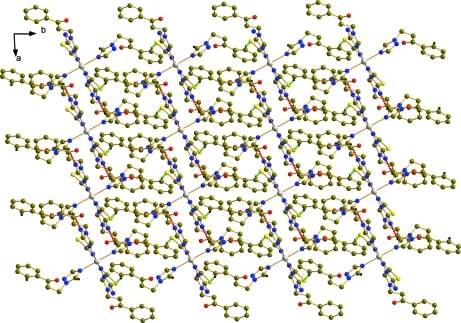
The two-dimensional sheet structure of the title compound, showing C—H···N and C—H···S hydrogen bongs as red and green dashed lines.

## supplementary crystallographic information

### Comment

Self-assembly processes directed by either hydrogen-bonding interactions or metal coordinations have been extensively utilized in crystal engineering to construct supramolecular systems with novel structures and properties due to their inherent strength and reliability (Braga *et al.*, 2003). Proper selection of metal ions and ligands with suitable functionalized groups is the key issue in designing and self-assembling of coordination supramolecules (Beatty, 2003). Recently, we have initiated a research program of synthesizing supramolecules based on pseudohalide and flexible ligand, which consists of a propanone unit substituted with an imidazole and a phenyl group (Guo & Cai, 2007). To further explore this series, we synthesized the title compound, a new Mn^II^ complex based on the mixed ligands, thiocyanate and 3-(1*H*-1,2,4-triazol-1-yl)-1-phenylpropan-1-one (*L*).

In the molecular structure (Fig. 1) of the mononuclear title complex, the Mn^II^ atom is six-coordinated by four monodentate *L* ligands, forming the equatorial plane and two N atoms from two monodentate NCS^-^ anions in the axial positions, displaying an MnN_6_ octahedral geometry. The triazol and phenyl rings in each of the ligands are not coplanar. The dihedral angels formed by the least-squares planes of the phenyl and triazole rings are 53.8 (2) and 69.6 (2)°. In the crystal, weak intermolecular C—H···N and C—H···S hydrogen bonds (Table 1) connect the complex molecules into a two-dimensional supramolecular sheet parallel to (0 0 1), as shown in Fig. 2.

### Experimental

MnCl_2_.4H_2_O (19.8 mg, 0.1 mmol), 3-(1*H*-1,2,4-triazol-1-yl)-1-phenylpropan-1-one (22.3 mg, 0.1 mmol) and NH_4_SCN (7.6 mg, 0.1 mmol) were mixed in a CH_3_CN/H_2_O (20 ml, v/v 1:1) solution with vigorous stirring for *ca* 30 min. The resulting solution was filtered and left to stand at room temperature. Colorless block crystals of the title compound suitable for X-ray analysis were obtained in a 60% yield by slow evaporation of the solvent over a period of one week. Analysis, calculated for C_46_H_44_MnN_14_O_4_S_2_: C 56.61, H 4.54, N 20.09%; found: C 56.45, H 4.43, N 20.12%.

### Refinement

Although all H atoms were visible in difference Fourier maps, they were finally placed in geometrically calculated positions and refined as riding atoms, with C—H = 0.93 (aromatic) and 0.97 (methylene) Å and with *U*_iso_(H) = 1.2*U*_eq_(C).

### Crystal data

**Table:**

[Mn(NCS)_2_(C_11_H_11_N_3_O)_4_]	*Z* = 1
*M**_r_* = 976.03	*F*(000) = 507
Triclinic, *P*1	*D*_x_ = 1.390 Mg m^−^^3^
Hall symbol: -P 1	Mo *K*α radiation, λ = 0.71073 Å
*a* = 7.9326 (17) Å	Cell parameters from 1514 reflections
*b* = 11.845 (3) Å	θ = 2.8–22.4°
*c* = 13.740 (3) Å	µ = 0.43 mm^−^^1^
α = 69.240 (3)°	*T* = 293 K
β = 75.417 (3)°	Block, colorless
γ = 81.686 (3)°	0.20 × 0.18 × 0.14 mm
*V* = 1166.1 (5) Å^3^	

### Data collection

**Table:**

Bruker APEXII CCD diffractometer	4075 independent reflections
Radiation source: fine-focus sealed tube	2840 reflections with *I* > 2σ(*I*)
graphite	*R*_int_ = 0.021
φ and ω scans	θ_max_ = 25.0°, θ_min_ = 2.0°
Absorption correction: multi-scan (*SADABS*; Sheldrick, 1996)	*h* = −8→9
*T*_min_ = 0.919, *T*_max_ = 0.942	*k* = −13→14
6410 measured reflections	*l* = −14→16

### Refinement

**Table:**

Refinement on *F*^2^	Primary atom site location: structure-invariant direct methods
Least-squares matrix: full	Secondary atom site location: difference Fourier map
*R*[*F*^2^ > 2σ(*F*^2^)] = 0.038	Hydrogen site location: inferred from neighbouring sites
*wR*(*F*^2^) = 0.089	H-atom parameters constrained
*S* = 1.06	*w* = 1/[σ^2^(*F*_o_^2^) + (0.0394*P*)^2^ + 0.0024*P*] where *P* = (*F*_o_^2^ + 2*F*_c_^2^)/3
4075 reflections	(Δ/σ)_max_ < 0.001
304 parameters	Δρ_max_ = 0.19 e Å^−^^3^
0 restraints	Δρ_min_ = −0.23 e Å^−^^3^

### Fractional atomic coordinates and isotropic or equivalent isotropic displacement parameters (Å^2^)

**Table:**

	*x*	*y*	*z*	*U*_iso_*/*U*_eq_	
Mn1	0.0000	0.0000	1.0000	0.03670 (15)	
S1	−0.34152 (10)	−0.18300 (7)	0.86875 (6)	0.0709 (2)	
O1	0.7108 (2)	0.12219 (16)	0.41612 (14)	0.0696 (5)	
O2	−0.2798 (3)	0.43808 (16)	0.66598 (16)	0.0763 (6)	
N1	0.2363 (2)	0.06526 (16)	0.87054 (14)	0.0429 (5)	
N2	0.4935 (2)	0.14559 (18)	0.78206 (15)	0.0532 (5)	
N3	0.4371 (2)	0.09195 (16)	0.72510 (14)	0.0425 (5)	
N4	−0.1390 (2)	0.18782 (15)	0.96560 (14)	0.0413 (5)	
N5	−0.2623 (3)	0.36007 (18)	0.99367 (16)	0.0532 (5)	
N6	−0.3215 (2)	0.34167 (17)	0.91665 (15)	0.0441 (5)	
N7	−0.1117 (3)	−0.03665 (19)	0.88320 (16)	0.0538 (5)	
C1	0.2858 (3)	0.0449 (2)	0.77873 (18)	0.0459 (6)	
H1	0.2231	0.0031	0.7550	0.055*	
C2	0.3686 (3)	0.1270 (2)	0.86777 (19)	0.0524 (6)	
H2	0.3708	0.1545	0.9230	0.063*	
C3	0.5430 (3)	0.0878 (2)	0.62302 (18)	0.0523 (6)	
H3A	0.4815	0.0482	0.5926	0.063*	
H3B	0.6517	0.0410	0.6337	0.063*	
C4	0.5813 (3)	0.2132 (2)	0.54720 (17)	0.0470 (6)	
H4A	0.6465	0.2512	0.5770	0.056*	
H4B	0.4721	0.2608	0.5398	0.056*	
C5	0.6839 (3)	0.2142 (2)	0.43876 (18)	0.0459 (6)	
C6	0.7487 (3)	0.3311 (2)	0.35991 (17)	0.0426 (6)	
C7	0.6996 (3)	0.4402 (2)	0.3773 (2)	0.0555 (7)	
H7	0.6264	0.4421	0.4413	0.067*	
C8	0.7584 (4)	0.5460 (2)	0.3002 (2)	0.0638 (7)	
H8	0.7225	0.6192	0.3121	0.077*	
C9	0.8682 (3)	0.5450 (3)	0.2070 (2)	0.0633 (7)	
H9	0.9077	0.6173	0.1558	0.076*	
C10	0.9208 (3)	0.4378 (3)	0.1883 (2)	0.0645 (8)	
H10	0.9964	0.4368	0.1247	0.077*	
C11	0.8608 (3)	0.3315 (2)	0.26470 (19)	0.0546 (7)	
H11	0.8963	0.2587	0.2520	0.066*	
C12	−0.2476 (3)	0.2397 (2)	0.90146 (18)	0.0429 (6)	
H12	−0.2693	0.2090	0.8524	0.051*	
C13	−0.1537 (3)	0.2650 (2)	1.02034 (19)	0.0506 (6)	
H13	−0.0918	0.2519	1.0729	0.061*	
C14	−0.4552 (3)	0.4253 (2)	0.8686 (2)	0.0570 (7)	
H14A	−0.5458	0.4428	0.9245	0.068*	
H14B	−0.5080	0.3868	0.8329	0.068*	
C15	−0.3844 (3)	0.5427 (2)	0.78923 (19)	0.0509 (6)	
H15A	−0.4804	0.6033	0.7789	0.061*	
H15B	−0.3055	0.5701	0.8188	0.061*	
C16	−0.2891 (3)	0.5332 (2)	0.6827 (2)	0.0509 (6)	
C17	−0.2102 (3)	0.6418 (2)	0.59709 (19)	0.0479 (6)	
C18	−0.2121 (3)	0.7519 (2)	0.6130 (2)	0.0607 (7)	
H18	−0.2654	0.7599	0.6789	0.073*	
C19	−0.1353 (4)	0.8486 (3)	0.5314 (2)	0.0715 (8)	
H19	−0.1376	0.9217	0.5427	0.086*	
C20	−0.0561 (4)	0.8392 (3)	0.4342 (2)	0.0702 (8)	
H20	−0.0043	0.9054	0.3797	0.084*	
C21	−0.0530 (4)	0.7320 (3)	0.4172 (2)	0.0764 (9)	
H21	0.0011	0.7249	0.3510	0.092*	
C22	−0.1298 (3)	0.6347 (3)	0.4978 (2)	0.0637 (7)	
H22	−0.1277	0.5623	0.4851	0.076*	
C23	−0.2068 (3)	−0.0969 (2)	0.87534 (17)	0.0438 (6)	

### Atomic displacement parameters (Å^2^)

**Table:**

	*U*^11^	*U*^22^	*U*^33^	*U*^12^	*U*^13^	*U*^23^
Mn1	0.0344 (3)	0.0396 (3)	0.0321 (3)	−0.0090 (2)	−0.0024 (2)	−0.0078 (2)
S1	0.0835 (5)	0.0780 (5)	0.0643 (5)	−0.0293 (4)	−0.0104 (4)	−0.0341 (4)
O1	0.0930 (14)	0.0559 (12)	0.0517 (12)	−0.0116 (10)	0.0070 (10)	−0.0209 (10)
O2	0.0934 (14)	0.0546 (12)	0.0837 (15)	−0.0020 (10)	−0.0071 (11)	−0.0356 (11)
N1	0.0423 (11)	0.0464 (12)	0.0356 (12)	−0.0131 (9)	−0.0017 (9)	−0.0090 (9)
N2	0.0519 (12)	0.0649 (14)	0.0437 (13)	−0.0276 (11)	0.0015 (10)	−0.0180 (11)
N3	0.0439 (11)	0.0465 (11)	0.0342 (11)	−0.0125 (9)	−0.0007 (9)	−0.0115 (9)
N4	0.0461 (12)	0.0391 (11)	0.0368 (11)	−0.0063 (9)	−0.0088 (9)	−0.0091 (9)
N5	0.0671 (14)	0.0482 (13)	0.0480 (13)	0.0001 (11)	−0.0184 (11)	−0.0179 (11)
N6	0.0433 (11)	0.0417 (12)	0.0448 (12)	−0.0033 (9)	−0.0111 (9)	−0.0099 (10)
N7	0.0488 (12)	0.0645 (14)	0.0515 (13)	−0.0091 (11)	−0.0147 (10)	−0.0184 (11)
C1	0.0445 (14)	0.0532 (15)	0.0390 (14)	−0.0148 (11)	−0.0065 (11)	−0.0113 (12)
C2	0.0595 (16)	0.0612 (16)	0.0376 (14)	−0.0266 (13)	0.0029 (12)	−0.0179 (12)
C3	0.0522 (15)	0.0573 (16)	0.0398 (14)	−0.0100 (12)	0.0057 (12)	−0.0152 (12)
C4	0.0445 (14)	0.0537 (15)	0.0349 (13)	−0.0037 (11)	−0.0013 (11)	−0.0098 (12)
C5	0.0429 (14)	0.0518 (15)	0.0395 (14)	−0.0015 (12)	−0.0063 (11)	−0.0134 (12)
C6	0.0405 (13)	0.0533 (15)	0.0322 (13)	−0.0052 (11)	−0.0080 (11)	−0.0110 (11)
C7	0.0627 (17)	0.0549 (17)	0.0412 (15)	−0.0019 (13)	−0.0043 (13)	−0.0120 (13)
C8	0.081 (2)	0.0497 (16)	0.0557 (18)	−0.0065 (14)	−0.0147 (16)	−0.0108 (14)
C9	0.0639 (18)	0.0649 (19)	0.0529 (18)	−0.0242 (15)	−0.0152 (14)	0.0000 (15)
C10	0.0645 (18)	0.082 (2)	0.0379 (15)	−0.0223 (16)	0.0035 (13)	−0.0123 (15)
C11	0.0609 (16)	0.0605 (17)	0.0402 (15)	−0.0095 (13)	−0.0027 (12)	−0.0174 (13)
C12	0.0432 (14)	0.0435 (14)	0.0420 (14)	−0.0090 (11)	−0.0056 (11)	−0.0143 (12)
C13	0.0664 (17)	0.0460 (15)	0.0431 (15)	−0.0026 (13)	−0.0194 (13)	−0.0142 (12)
C14	0.0443 (15)	0.0577 (17)	0.0664 (18)	0.0029 (13)	−0.0162 (13)	−0.0167 (14)
C15	0.0532 (15)	0.0448 (14)	0.0529 (16)	0.0092 (12)	−0.0183 (13)	−0.0141 (13)
C16	0.0512 (15)	0.0448 (15)	0.0614 (17)	0.0087 (12)	−0.0222 (13)	−0.0208 (14)
C17	0.0501 (14)	0.0457 (15)	0.0496 (15)	0.0125 (12)	−0.0206 (12)	−0.0172 (12)
C18	0.0799 (19)	0.0510 (16)	0.0476 (16)	0.0053 (14)	−0.0106 (14)	−0.0177 (14)
C19	0.090 (2)	0.0491 (17)	0.069 (2)	0.0007 (15)	−0.0149 (18)	−0.0154 (16)
C20	0.0660 (18)	0.068 (2)	0.0579 (19)	0.0036 (15)	−0.0109 (15)	−0.0029 (16)
C21	0.071 (2)	0.088 (2)	0.0564 (19)	0.0139 (18)	−0.0056 (16)	−0.0210 (18)
C22	0.0677 (18)	0.0635 (18)	0.0621 (19)	0.0128 (15)	−0.0150 (15)	−0.0294 (16)
C23	0.0484 (15)	0.0493 (15)	0.0325 (13)	0.0019 (12)	−0.0090 (11)	−0.0139 (11)

### Geometric parameters (Å, °)

**Table:**

Mn1—N7	2.207 (2)	C7—C8	1.375 (3)
Mn1—N1	2.2452 (17)	C7—H7	0.9300
Mn1—N4	2.2796 (18)	C8—C9	1.358 (4)
S1—C23	1.619 (3)	C8—H8	0.9300
O1—C5	1.212 (3)	C9—C10	1.370 (4)
O2—C16	1.216 (3)	C9—H9	0.9300
N1—C1	1.320 (3)	C10—C11	1.378 (3)
N1—C2	1.349 (3)	C10—H10	0.9300
N2—C2	1.307 (3)	C11—H11	0.9300
N2—N3	1.352 (2)	C12—H12	0.9300
N3—C1	1.323 (3)	C13—H13	0.9300
N3—C3	1.456 (3)	C14—C15	1.510 (3)
N4—C12	1.320 (3)	C14—H14A	0.9700
N4—C13	1.352 (3)	C14—H14B	0.9700
N5—C13	1.315 (3)	C15—C16	1.504 (3)
N5—N6	1.353 (2)	C15—H15A	0.9700
N6—C12	1.326 (3)	C15—H15B	0.9700
N6—C14	1.459 (3)	C16—C17	1.490 (3)
N7—C23	1.158 (3)	C17—C22	1.380 (3)
C1—H1	0.9300	C17—C18	1.393 (3)
C2—H2	0.9300	C18—C19	1.374 (4)
C3—C4	1.503 (3)	C18—H18	0.9300
C3—H3A	0.9700	C19—C20	1.361 (4)
C3—H3B	0.9700	C19—H19	0.9300
C4—C5	1.504 (3)	C20—C21	1.367 (4)
C4—H4A	0.9700	C20—H20	0.9300
C4—H4B	0.9700	C21—C22	1.373 (4)
C5—C6	1.490 (3)	C21—H21	0.9300
C6—C7	1.380 (3)	C22—H22	0.9300
C6—C11	1.383 (3)		
			
N7—Mn1—N7^i^	180.0	C8—C7—H7	119.9
N7—Mn1—N1^i^	91.32 (7)	C6—C7—H7	119.9
N7^i^—Mn1—N1^i^	88.68 (7)	C9—C8—C7	120.8 (3)
N7—Mn1—N1	88.68 (7)	C9—C8—H8	119.6
N7^i^—Mn1—N1	91.32 (7)	C7—C8—H8	119.6
N1^i^—Mn1—N1	180.0	C8—C9—C10	120.1 (3)
N7—Mn1—N4^i^	89.00 (7)	C8—C9—H9	119.9
N7^i^—Mn1—N4^i^	91.00 (7)	C10—C9—H9	119.9
N1^i^—Mn1—N4^i^	93.23 (6)	C9—C10—C11	119.4 (2)
N1—Mn1—N4^i^	86.77 (6)	C9—C10—H10	120.3
N7—Mn1—N4	91.00 (7)	C11—C10—H10	120.3
N7^i^—Mn1—N4	89.00 (7)	C10—C11—C6	121.2 (2)
N1^i^—Mn1—N4	86.77 (6)	C10—C11—H11	119.4
N1—Mn1—N4	93.23 (6)	C6—C11—H11	119.4
N4^i^—Mn1—N4	180.000 (1)	N4—C12—N6	110.5 (2)
C1—N1—C2	102.08 (18)	N4—C12—H12	124.7
C1—N1—Mn1	127.56 (15)	N6—C12—H12	124.7
C2—N1—Mn1	130.23 (15)	N5—C13—N4	115.0 (2)
C2—N2—N3	102.28 (18)	N5—C13—H13	122.5
C1—N3—N2	109.70 (18)	N4—C13—H13	122.5
C1—N3—C3	129.7 (2)	N6—C14—C15	112.91 (19)
N2—N3—C3	120.58 (18)	N6—C14—H14A	109.0
C12—N4—C13	102.41 (19)	C15—C14—H14A	109.0
C12—N4—Mn1	129.36 (15)	N6—C14—H14B	109.0
C13—N4—Mn1	127.34 (15)	C15—C14—H14B	109.0
C13—N5—N6	102.19 (19)	H14A—C14—H14B	107.8
C12—N6—N5	109.89 (19)	C16—C15—C14	113.7 (2)
C12—N6—C14	130.0 (2)	C16—C15—H15A	108.8
N5—N6—C14	120.02 (19)	C14—C15—H15A	108.8
C23—N7—Mn1	143.02 (18)	C16—C15—H15B	108.8
N1—C1—N3	110.6 (2)	C14—C15—H15B	108.8
N1—C1—H1	124.7	H15A—C15—H15B	107.7
N3—C1—H1	124.7	O2—C16—C17	120.4 (2)
N2—C2—N1	115.3 (2)	O2—C16—C15	120.1 (2)
N2—C2—H2	122.3	C17—C16—C15	119.5 (2)
N1—C2—H2	122.3	C22—C17—C18	117.7 (2)
N3—C3—C4	110.74 (19)	C22—C17—C16	119.6 (2)
N3—C3—H3A	109.5	C18—C17—C16	122.7 (2)
C4—C3—H3A	109.5	C19—C18—C17	120.1 (3)
N3—C3—H3B	109.5	C19—C18—H18	119.9
C4—C3—H3B	109.5	C17—C18—H18	119.9
H3A—C3—H3B	108.1	C20—C19—C18	121.1 (3)
C3—C4—C5	112.8 (2)	C20—C19—H19	119.4
C3—C4—H4A	109.0	C18—C19—H19	119.4
C5—C4—H4A	109.0	C19—C20—C21	119.6 (3)
C3—C4—H4B	109.0	C19—C20—H20	120.2
C5—C4—H4B	109.0	C21—C20—H20	120.2
H4A—C4—H4B	107.8	C20—C21—C22	120.0 (3)
O1—C5—C6	121.1 (2)	C20—C21—H21	120.0
O1—C5—C4	120.6 (2)	C22—C21—H21	120.0
C6—C5—C4	118.3 (2)	C21—C22—C17	121.5 (3)
C7—C6—C11	118.2 (2)	C21—C22—H22	119.3
C7—C6—C5	122.6 (2)	C17—C22—H22	119.3
C11—C6—C5	119.2 (2)	N7—C23—S1	178.0 (2)
C8—C7—C6	120.3 (2)		
			
N7—Mn1—N1—C1	−17.61 (19)	O1—C5—C6—C7	170.4 (2)
N7^i^—Mn1—N1—C1	162.39 (19)	C4—C5—C6—C7	−8.7 (3)
N4^i^—Mn1—N1—C1	71.47 (19)	O1—C5—C6—C11	−8.7 (3)
N4—Mn1—N1—C1	−108.53 (19)	C4—C5—C6—C11	172.2 (2)
N7—Mn1—N1—C2	167.2 (2)	C11—C6—C7—C8	1.4 (4)
N7^i^—Mn1—N1—C2	−12.8 (2)	C5—C6—C7—C8	−177.7 (2)
N4^i^—Mn1—N1—C2	−103.7 (2)	C6—C7—C8—C9	−1.4 (4)
N4—Mn1—N1—C2	76.3 (2)	C7—C8—C9—C10	0.5 (4)
C2—N2—N3—C1	−0.3 (3)	C8—C9—C10—C11	0.3 (4)
C2—N2—N3—C3	−177.8 (2)	C9—C10—C11—C6	−0.1 (4)
N7—Mn1—N4—C12	2.65 (19)	C7—C6—C11—C10	−0.7 (4)
N7^i^—Mn1—N4—C12	−177.35 (19)	C5—C6—C11—C10	178.5 (2)
N1^i^—Mn1—N4—C12	−88.62 (19)	C13—N4—C12—N6	0.3 (2)
N1—Mn1—N4—C12	91.38 (19)	Mn1—N4—C12—N6	170.01 (13)
N7—Mn1—N4—C13	169.91 (18)	N5—N6—C12—N4	−0.2 (2)
N7^i^—Mn1—N4—C13	−10.09 (18)	C14—N6—C12—N4	−176.4 (2)
N1^i^—Mn1—N4—C13	78.64 (18)	N6—N5—C13—N4	0.3 (3)
N1—Mn1—N4—C13	−101.36 (18)	C12—N4—C13—N5	−0.4 (3)
C13—N5—N6—C12	0.0 (2)	Mn1—N4—C13—N5	−170.35 (15)
C13—N5—N6—C14	176.6 (2)	C12—N6—C14—C15	−107.7 (3)
N1^i^—Mn1—N7—C23	−27.0 (3)	N5—N6—C14—C15	76.5 (3)
N1—Mn1—N7—C23	153.0 (3)	N6—C14—C15—C16	77.9 (3)
N4^i^—Mn1—N7—C23	66.2 (3)	C14—C15—C16—O2	2.3 (3)
N4—Mn1—N7—C23	−113.8 (3)	C14—C15—C16—C17	−179.2 (2)
C2—N1—C1—N3	−0.3 (3)	O2—C16—C17—C22	1.5 (4)
Mn1—N1—C1—N3	−176.47 (14)	C15—C16—C17—C22	−177.0 (2)
N2—N3—C1—N1	0.4 (3)	O2—C16—C17—C18	−177.9 (2)
C3—N3—C1—N1	177.6 (2)	C15—C16—C17—C18	3.6 (3)
N3—N2—C2—N1	0.2 (3)	C22—C17—C18—C19	−0.2 (4)
C1—N1—C2—N2	0.0 (3)	C16—C17—C18—C19	179.2 (2)
Mn1—N1—C2—N2	176.10 (16)	C17—C18—C19—C20	−0.2 (4)
C1—N3—C3—C4	125.4 (2)	C18—C19—C20—C21	0.2 (4)
N2—N3—C3—C4	−57.7 (3)	C19—C20—C21—C22	0.1 (4)
N3—C3—C4—C5	−177.45 (19)	C20—C21—C22—C17	−0.5 (4)
C3—C4—C5—O1	8.0 (3)	C18—C17—C22—C21	0.5 (4)
C3—C4—C5—C6	−172.95 (19)	C16—C17—C22—C21	−178.9 (2)

Symmetry codes: (i) −*x*, −*y*, −*z*+2.

### Hydrogen-bond geometry (Å, °)

**Table:**

*D*—H···*A*	*D*—H	H···*A*	*D*···*A*	*D*—H···*A*
C12—H12···N2^ii^	0.93	2.62	3.436 (3)	146
C18—H18···S1^iii^	0.93	2.82	3.725 (3)	164

Symmetry codes: (ii) *x*−1, *y*, *z*; (iii) *x*, *y*+1, *z*.
